# Investigating Data Diversity and Model Robustness of AI Applications in Palliative Care and Hospice: Protocol for Scoping Review

**DOI:** 10.2196/56353

**Published:** 2024-10-08

**Authors:** Selen Bozkurt, Soraya Fereydooni, Irem Kar, Catherine Diop Chalmers, Sharon L Leslie, Ravi Pathak, Anne Walling, Charlotta Lindvall, Karl Lorenz, Tammie Quest, Karleen Giannitrapani, Dio Kavalieratos

**Affiliations:** 1 Department of Biomedical Informatics Emory University Atlanta, GA United States; 2 Division of Palliative Medicine Department of Family and Preventive Medicine Emory University Atlanta, GA United States; 3 Center for Ethics Emory University Atlanta Georgia; 4 Yale School of Medicine New Haven, CT United States; 5 Department of Biostatistics Ankara University Ankara Turkey; 6 Winship Cancer Institute Emory University Atlanta, GA United States; 7 Woodruff Health Sciences Center Library Emory University Atlanta, GA United States; 8 Department of Medicine Veterans Affairs Greater Los Angeles Health System Los Angeles, CA United States; 9 Department of Medicine University of California Los Angeles, CA United States; 10 Harvard Medical School Boston, MA United States; 11 Department of Informatics and Analytics Dana-Farber Cancer Institute Boston, MD United States; 12 Department of Medicine Brigham and Women’s Hospital Boston, MD United States; 13 Department of Psychosocial Oncology and Palliative Care Dana-Farber Cancer Institute Boston, MD United States; 14 Primary Care and Population Health School of Medicine Stanford University Stanford, CA United States; 15 Center for Innovation to Implementation (Ci2i) Veterans Affairs Palo Alto Health Care System Palo Alto, CA United States

**Keywords:** palliative care, artificial intelligence, ethical frameworks, AI, data diversity, model robustness, decision support, clinical settings, end-of-life care, hospice environments, hospice, methodology, thematic analysis, dissemination

## Abstract

**Background:**

Artificial intelligence (AI) has become a pivotal element in health care, leading to significant advancements across various medical domains, including palliative care and hospice services. These services focus on improving the quality of life for patients with life-limiting illnesses, and AI’s ability to process complex datasets can enhance decision-making and personalize care in these sensitive settings. However, incorporating AI into palliative and hospice care requires careful examination to ensure it reflects the multifaceted nature of these settings.

**Objective:**

This scoping review aims to systematically map the landscape of AI in palliative care and hospice settings, focusing on the data diversity and model robustness. The goal is to understand AI’s role, its clinical integration, and the transparency of its development, ultimately providing a foundation for developing AI applications that adhere to established ethical guidelines and principles.

**Methods:**

Our scoping review involves six stages: (1) identifying the research question; (2) identifying relevant studies; (3) study selection; (4) charting the data; (5) collating, summarizing, and reporting the results; and (6) consulting with stakeholders. Searches were conducted across databases including MEDLINE through PubMed, Embase.com, IEEE Xplore, ClinicalTrials.gov, and Web of Science Core Collection, covering studies from the inception of each database up to November 1, 2023. We used a comprehensive set of search terms to capture relevant studies, and non-English records were excluded if their abstracts were not in English. Data extraction will follow a systematic approach, and stakeholder consultations will refine the findings.

**Results:**

The electronic database searches conducted in November 2023 resulted in 4614 studies. After removing duplicates, 330 studies were selected for full-text review to determine their eligibility based on predefined criteria. The extracted data will be organized into a table to aid in crafting a narrative summary. The review is expected to be completed by May 2025.

**Conclusions:**

This scoping review will advance the understanding of AI in palliative care and hospice, focusing on data diversity and model robustness. It will identify gaps and guide future research, contributing to the development of ethically responsible and effective AI applications in these settings.

**International Registered Report Identifier (IRRID):**

DERR1-10.2196/56353

## Introduction

### Background

Artificial Intelligence (AI) has become a pivotal element in health care at large, leading to significant advancements across various medical domains, including palliative care and hospice services [1,2]. Palliative care and hospice services focus on improving the quality of life for patients with life-limiting illnesses: palliative care provides relief from the symptoms and stress of serious illness, while hospice care offers comfort for patients in the final stages of a terminal illness. AI’s ability to process complex datasets can enhance decision-making and personalize care in these sensitive settings. Recent studies have demonstrated the potential of AI to improve patient outcomes in palliative care by predicting symptom trajectories, optimizing pain management, and identifying patients who may benefit from hospice services [1,2]. For instance, AI models can analyze electronic health records to detect patterns that may indicate a decline in a patient’s condition, allowing for timely interventions [3].

Despite its potential, incorporating AI into palliative and hospice care requires careful examination to ensure it reflects the multifaceted nature of these settings. AI models must be robust enough to withstand the complexities of palliative care environments. Moreover, the robustness of AI models must be scrutinized to withstand the complex dynamics of palliative care environments. There is growing advocacy for ethical AI applications that can sensitively meet the unique needs of patients receiving palliative care, such as personalized care plans and real-time monitoring for complex symptom management while safeguarding against the amplification of existing biases within health care data [3-5]. As such, there is a pressing need to evaluate the representativeness and robustness of AI in these settings critically.

A few reviews have explored the application of AI in palliative and hospice care, but these reviews often focus on specific types of diseases or the general benefits of AI applications without delving into detailed aspects of model performance, bias, or robustness [1,2,6].

This gap highlights the need for a comprehensive review that critically examines these important factors in the context of palliative and hospice care. This review aims to address this gap by systematically mapping the landscape of AI applications in palliative care and hospice settings, focusing on the diversity of data in AI models and their robustness in clinical integration. By expanding the literature and including recent studies and key papers on AI applications in palliative care and hospice, we aim to provide a comprehensive overview that will guide future research and contribute to the development of effective and ethically responsible AI applications in these sensitive settings.

### Goals of the Review

Our scoping review aims to systematically map the landscape of AI in palliative care and hospice settings by focusing on the diversity of data in AI models and the robustness of the models. We seek to understand AI’s role, the extent of its clinical integration, and the transparency of its development. We believe this comprehensive overview will provide the foundation for developing AI applications that adhere to established ethical guidelines and principles, ensuring fairness, transparency, and accountability.

## Methods

### Design

#### Overview

Our scoping review was guided by Arksey and O’Malley’s [7] framework and Joanna Briggs’ manual for scoping reviews [8], which consists of six main stages: (1) identifying the research question; (2) identifying relevant studies; (3) study selection; (4) charting the data; (5) collating, summarizing, and reporting the results; and (6) consulting with stakeholders to validate findings and enhance the comprehensiveness of the review. This scoping review has been registered in the Open Science Framework (OSF) database [9]. For reporting, we will use PRISMA-S (Preferred Reporting Items for Systematic Reviews and Meta-Analyses–Statements) for our searches and PRISMA-ScR (PRISMA-Extension for Scoping Reviews) for the review itself [10].

#### Stage 1: Identifying the Research Question

The interdisciplinary research team, including experts in palliative care, informatics, data science, and public health, initiated the scoping review with an informal prescreening of literature from MEDLINE through PubMed and various gray literature sources. With the guidance of an information specialist in scoping reviews, keywords were identified and used to navigate the research terrain. Contributions from the team, reflecting their diverse fields of expertise, along with the analysis of 5 key papers and 5 previous reviews, shaped the development of broad research questions. Through discussion, the team generated the research questions stated below.

#### Primary Research Questions

1. Research question 1: use in hospice and palliative care: in what ways are AI used to identify patients for hospice and palliative care or to measure or improve the care quality for patients receiving hospice and palliative care?
2. Research question 2: data diversity: in the development of AI models for palliative and hospice care, what strategies are used to incorporate diverse demographic and clinical profiles? In addition, which methodologies ensure the representativeness of the datasets used?
3. Research question 3: model robustness: what frameworks and best practice guidelines inform the development of AI models within palliative and hospice care?
4. Research question 4: bias assessment: how is bias detection and mitigation systematically integrated and what ethical guidelines are in place?

#### Secondary Research Questions

1. Research question 5: applications and outcomes: what specific tasks and outcomes are targeted by AI technologies in palliative care and hospice settings?
2. Research question 6: research trajectory: what are the emergent directions and identified literature gaps in AI for palliative and hospice care?

#### Stage 2: Identifying Relevant Studies

##### Peer-Reviewed Literature

The databases MEDLINE through PubMed, Embase.com, IEEE Xplore, ClinicalTrials.gov, and Web of Science Core Collection were used to search for peer-reviewed literature. These searches were developed by an information specialist. Our search strategy included studies from the inception of each database up to November 1, 2023. This approach was taken to ensure comprehensive coverage and to capture the full breadth of relevant literature on AI applications in palliative care and hospice settings. Non-English records were excluded from further processing if their abstracts were not in English. The search terms and queries, detailed in Multimedia Appendix 1, along with the inclusion and exclusion criteria outlined in Textbox 1, were formulated after reviewing relevant publications and consulting with coauthors and information specialists.

Specifically, terms relating to “advanced disease” were not included in the search strategy. After thorough discussion, it was concluded that including “advanced disease” is too diffuse a term and could lead to an overwhelming and unmanageable volume of data, complicating the review process. In a similar vein, terms related to clinical decision support (CDS) were also intentionally excluded from our search strategy. This exclusion was based on the finding that a substantial number of CDS methods do not integrate AI technologies. However, this does not mean CDS studies are excluded. Any CDS studies using AI technologies will be identified through our AI-related search terms. Our primary focus was on AI techniques rather than specific names of tools. By emphasizing AI techniques, our goal was to ensure that our review concentrated on the fundamental role of AI in palliative care and hospice settings. We believe that this strategy will enable us to focus on the integration and use of AI as a distinct technological paradigm in these areas, as opposed to a broader examination of CDS tools, many of which may not use AI technologies.

Our research will encompass studies conducted across various international health systems. This inclusive approach is chosen to capture a diverse range of health care practices and methodologies globally, which are vital for providing a comprehensive understanding and guiding future clinical quality improvements and research in the field of palliative and hospice care.

**Inclusion and exclusion criteria for the scoping review.**

**Phenomenon of interest**

- Artificial intelligence applications in palliative care and hospice

**Inclusion criteria**

- Study type:
  Observational studies (cohort, case-control, and cross-sectional)
  Experimental studies (randomized controlled trials, nonrandomized, and experimental)
  Other study designs: (qualitative, case series, diagnostic test accuracy, clinical prediction rule, and economic evaluations)
  Gray literature: conference papers and PhD theses
- Population type:
  Patients aged 18 and older: those with severe life-limiting illness, and patients who are already enrolled in palliative care or hospice settings
  Studies that reported recruiting both adults and children will be included only if the results were stratified by age group
- Artificial intelligence model criteria: the study must either:
  Propose or use an artificial intelligence model that uses a training set to learn its model parameters, or
  Implement a pre-established artificial intelligence model, focusing on deployment and practical application in the field
- Setting:
  All care settings (ie, inpatient and outpatient) within palliative and hospice care
- Other:
  Available electronically in full text
  Articles in English
  Studies must explicitly state their aim to identify patients for hospice and palliative care or to measure or improve the care quality for patients receiving hospice and palliative care.

**Exclusion criteria**

- Study type:
  Conference abstracts with no full text
  Case studies, editorials, reports, and reviews
  Books and book chapters
  Letters to editors and perspectives
- Other
  Any study not directly targeting palliative care, as determined by the absence of specific search terms (Multimedia Appendix 1). Studies mentioning these groups only as a subgroup or solely using the term “Inoperable or Incurable” without explicit reference to palliative care will also be excluded.
  Studies that solely used traditional, rule-based algorithms without any artificial intelligence components

##### Gray Literature

In our research on AI applications in palliative care and hospice, we used Web of Science to identify both peer-reviewed and gray literature. Conference abstracts and proceedings were located through Embase.com and Web of Science. Theses and dissertations were collected through ProQuest Theses & Dissertations Global. Preprints from platforms like ArXiv were excluded as they can be withdrawn or significantly revised after initial posting, affecting the stability and reliability of the information. In addition, excluding these preprints avoids duplication, as many are eventually published in peer-reviewed journals.

All peer-reviewed and gray literature results will be downloaded into EndNote X20 (Clarivate Analytics) and imported into the web-based systematic review software (version 2.0; Covidence) for review. The expert authors’ committee (RP, AW, KG, and DK) will also be asked to identify other potentially relevant peer-reviewed and gray literature materials not identified through previous search strategies (ie, “hand-searched” articles). Duplicates will be removed using EndNote and Covidence software to ensure a clean and accurate dataset for review. Covidence software will also be used for screening to facilitate blinding and streamline the review process.

#### Stage 3: Study Selection

A screening guide developed by the reviewers (SB and SF) will be used to determine if the inclusion and exclusion criteria have been met (Textbox 1). Feedback will be obtained from the coauthors (KG and DK) and palliative care experts (RP, AW, CL, KL, and TQ) while developing the screening guide. The 4 reviewers (SB, SF, IK, and CD) will then independently pilot-test this guide on the first 100 abstracts. The first 100 abstracts were selected using the “most relevant articles” option provided by Covidence software. Agreement rates will be evaluated after the initial 15 and again after 100 abstracts, with discussions to follow and adjustments to the inclusion or exclusion criteria made as necessary. Any discrepancies in study selection will be resolved through consensus. To ensure the appropriateness of the selections, an example of both an included and an excluded article will be presented to the project team. Subsequently, all remaining articles will be independently screened by at least 2 reviewers using the guide, focusing on titles and abstracts for their relevance to “artificial intelligence,” “palliative care and hospice,” and the general inclusion criteria. The reviewers will meet at the beginning, middle, and end of the screening process to discuss any challenges and ensure consistent application of the criteria.

After this initial phase, the full texts of articles that pass the initial screening will be reviewed at least by 2 independent reviewers to assess their relevance to the primary research questions of the study. If disagreements among reviewers cannot be resolved through discussion, the principal investigator (SB) will make the final decisions. Regular check-in meetings will be scheduled to discuss results and resolve any discrepancies, ensuring a comprehensive and systematic approach to study selection. Reasons for the exclusion of full-text papers will be recorded using the PRISMA 2020 flow diagram.

#### Stage 4: Charting the Data

We will design a data charting form in Covidence, specifically structured to accommodate each primary research question. These questions are a combination of those from the Minimum Information for Clinical Artificial Intelligence Modeling (MI-CLAIM) checklist [11] and additional ones formulated by our authorship team. We chose MI-CLAIM due to its comprehensive coverage and practicality for our scoping review. AI-related studies benefit from several reporting guidelines, thanks to pioneering efforts by the SPIRIT-AI (Standard Protocol Items: Recommendations for Interventional Trials-Artificial Intelligence) [12,13] and CONSORT-AI (Consolidated Standards of Reporting Trials–Artificial Intelligence) [12] extensions committees. Similarly, for review studies, the PRISMA-AI Steering Committee is developing an AI-specific implementation of PRISMA guidelines, which will be used in future studies to further standardize AI research reporting [14].

This form’s sample layout is depicted in Textbox 2. To validate these forms for both academic and gray literature, our reviewers (SB and CD) will initially chart data from 10 included sources independently. Following this pilot phase, once interrater reliability is confirmed, these forms will then be made available to all team members for use. For a portion, specifically 20%, of the included academic and gray literature sources, the data extraction will be verified by a second reviewer. Recognizing that data charting is a dynamic process, it is anticipated that the team might modify aspects of the forms to ensure they accurately reflect the findings of the included articles. After achieving consistency and finalizing the pilot-tested forms, data from each included full-text article will be charted by 1 member of the research team.

**Data that will be charted.**

- **Article information**
  Title
  Author
  Year
- **Study context**
  The clinical problem in which the model will be used is clearly detailed in the paper (the questions are derived from the Minimum Information for Clinical Artificial Intelligence Modeling checklist; yes or no)
  Study design: eg, randomized controlled trial, cross-sectional, and qualitative
  Clinical condition or disease: eg, coronary artery disease, chronic obstructive pulmonary disease, and Alzheimer disease
  Clinical setting: eg, hospice, inpatient hospice, and outpatient palliative clinic
- **Data**
  The origin of the data is described, and the original format is detailed in the paper (the questions are derived from the Minimum Information for Clinical Artificial Intelligence Modeling checklist; yes or no)
  Data Source: eg, electronic health records, publicly available data, and study-specific data; if MIMIC (Medical Information Mart for Intensive Care), specify “MIMIC”
  Data modality: eg, images, audio recordings, and multimodal
  Data volume: text entry for number of records and patients
  The characteristics of the cohorts (training and test sets) are detailed in the text (the questions are derived from the Minimum Information for Clinical Artificial Intelligence Modeling checklist; yes or no). If yes, then (1) age range (min-max), (2) gender distribution (female percentage), (3) race distribution (White percentage), (4) ethnicity distribution (Hispanic percentage), (5) does the article report socioeconomic status as demographic information of the study population? (yes or no), and (6) does the article report insurance as demographic information of the study population? (yes or no)
  The cohorts (training and test sets) are shown to be representative of real-world clinical settings (the questions are derived from the Minimum Information for Clinical Artificial Intelligence Modeling checklist; yes or no). If yes then, what strategies or frameworks are reported to ensure the dataset’s representativeness and heterogeneity in the artificial intelligence study? (text entry)
- **Artificial intelligence**
  Artificial intelligence outcome: eg, survival, disease risk, and metastasis
  Have any specific design, development, or evaluation frameworks or guidelines been used in the artificial intelligence technology’s lifecycle? If yes, please provide details (yes or no; text entry)
  Reported accuracy: text entry for the highest metric, prioritize *F*_1_-score and area under the curve (AUC)
  Evaluation: eg, in silico (proof of concept), offline (silent and shadow), safety and utility, small-scale (early live clinical), safety and effectiveness, large-scale (comparative perspective), and postmarket surveillance
- **Bias identification and attribution**
  Has a biased evaluation been conducted or referenced within the study? (yes or no). If yes then, what methods, if any, have been proposed or implemented to address identified biases? (text entry)
  A discussion of the reliability and robustness of the model as the underlying data distribution shifts (eg, changes in patient demographics, treatment guidelines, or health care protocols over time) is included (the questions are derived from the Minimum Information for Clinical Artificial Intelligence Modeling checklist; yes or no)
- **Other**
  Data publicly available: yes or no
  Model open source: yes or no
  Ethical approval mentioned: yes or no
  Informed consent mentioned: yes or no
  Funding source: who funded the research or development of the artificial intelligence model? Or any report of conflict of interest (text entry)

#### Stage 5: Collating, Summarizing, and Reporting the Results

Data charting will be the initial step in summarizing our research findings. In this process, we will meticulously record key information for each study, such as the article title, author or authors, year of publication, and the study’s objectives. Recognizing the practices in other AI reviews, we will also document technology-specific details, including the model’s task, output, its development stage, the data sources used, and the methods used for evaluation.

To effectively synthesize the findings from multiple studies on AI applications in palliative care and hospice settings, we will use a framework analysis approach. This method involves systematically categorizing and organizing data according to predefined themes. Initially, we will familiarize ourselves with the data by reading and rereading the studies, noting key findings. A thematic framework will be developed based on our research objectives, including themes such as the model’s task, output, development stage, data sources, and evaluation methods. We will then index the data according to this framework, systematically coding and categorizing relevant information. Charts or matrices will be created to organize the indexed data, allowing us to map out patterns and explore relationships between themes. Through iterative discussions, we will review and refine the framework and charts, ensuring the robustness of our analysis. This approach will provide a comprehensive understanding of AI applications in palliative care and hospice, highlighting commonalities, differences, and research gaps to guide future developments in this field.

Depending on the nature of the results obtained, we may also create visual representations to aid in clearer communication and understanding of the data. These visuals can include charts or graphs, providing an accessible way to grasp complex patterns and insights derived from the research. This multifaceted approach to data charting and analysis is aimed at producing a comprehensive and nuanced understanding of AI applications in palliative care and hospice settings.

#### Stage 6: Consultation

To incorporate wider perspectives, we will share the preliminary summary of our findings with teams of authors through video calls and emails to assess the alignment of identified themes with their expertise and to highlight any missing themes. Structured presentations and discussions will follow with the senior authorship team, comprising palliative care physicians and researchers, and health services and organizational behavior experts, to explore future research directions. Feedback from these sessions will be systematically reviewed and integrated into the analysis, refining the themes and ensuring comprehensive coverage. A final round of consultation will review the refined themes and overall findings to ensure consensus. The integration of consultation feedback will be documented, highlighting its influence on the final analysis and presentation of results.

### Ethical Considerations

Ethical approval was not required because only literature will be evaluated without accessing identifiable source data.

## Results

In November 2023, our electronic database searches resulted in 4614 studies. After the removal of 1124 duplicates, we selected 330 studies for full-text review to determine their eligibility and inclusion in our scoping review based on predefined criteria. Following this review, we will finalize the set of studies for data extraction. The extracted data will be methodically organized into a table to aid in crafting a narrative summary. Our primary method of presenting these findings will be through a scoping review publication. The entire process, encompassing the screening of titles and abstracts, charting of data, and subsequent stages of the scoping review, is projected to be completed by May 2025. This timeline also includes the execution of dissemination activities, such as a symposium and a briefing paper.

## Discussion

### Principal Findings

The findings of this review are set to significantly advance our understanding of AI in palliative care and hospice, with a particular emphasis on the challenges of data diversity and model robustness. A notable concern is the prevalence of data diversity issues and biases within AI models in these settings. Despite these challenges, there seems to be a lack of uniformly applied, robust frameworks to address them effectively, raising concerns about potential disparities in the effectiveness and equity of AI applications in palliative and hospice care. This underscores the urgent need for more stringent ethical and operational frameworks.

A key strength of our review lies in its interdisciplinary nature, offering insights into the use of AI in palliative and hospice care from societal, legal, ethical, and technical perspectives. Our methodology, grounded in a scoping review framework and encompassing interdisciplinary databases, is thorough. The diverse team of researchers involved in this study will analyze and interpret findings, which are expected to stimulate further discussions and guide future research, particularly focusing on AI applications for vulnerable adults requiring palliative and hospice care.

### Limitations

One limitation of our study is its exclusion of non-AI (rule-based) algorithms, narrowing our focus to AI-driven technologies and omitting insights from traditional systems in palliative and hospice care. This could limit our understanding of the full technological evolution in these fields. However, the current trend towards AI-driven models justifies this focus, as it reflects the growing importance and anticipated future dominance of AI in health care. Another limitation of our study is the inclusion of only English-language literature, which may lead to the omission of relevant non-English studies, potentially introducing language bias and limiting the comprehensiveness of our review. In our upcoming full-text review, we will document the number of non-English records identified and screened. For non-English records with abstracts in English, we will assess their relevance and note those not further processed despite their relevance at the title and abstract screening stage to address potential biases introduced by excluding non-English evidence. We also acknowledge the potential limitation of not identifying all possible search terms and synonyms for palliative care. Despite our comprehensive search strategy, some relevant studies may have been missed. Future research should refine and expand search terms for more exhaustive coverage.

### Conclusions

In light of the increased awareness of bias in AI, we anticipate that newer studies will more comprehensively address data representativeness and diversity, likely showcasing a commitment to ethical AI practices in health care. To our best knowledge, this is the first scoping review to explore the data diversity and robustness of AI models specifically in palliative care and hospice settings. Our protocol outlines not just our search strategy but also the detailed process for synthesizing literature in this clinical domain catering to a vulnerable population. Through this review, we aim to provide valuable insights, guide future research, and contribute to the development of ethically responsible and effective AI applications in palliative care and hospice environments.

## Appendix

Multimedia Appendix 1 Keywords and search strategies.

## Abbreviations

AI: artificial intelligence
CDI: clinical decision support
CONSORT-AI: Consolidated Standards of Reporting Trials–Artificial Intelligence
MI-CLAIM: Minimum Information for Clinical Artificial Intelligence Modeling
OSF: Open Science Framework
PRISMA-AI: Preferred Reporting Items for Systematic Reviews and Meta-Analyses for Artificial Intelligence
PRISMA-S: Preferred Reporting Items for Systematic Reviews and Meta-Analyses–Statement
PRISMA-ScR: Preferred Reporting Items for Systematic Reviews and Meta-Analyses Extension for Scoping Reviews
SPIRIT-AI: Standard Protocol Items: Recommendations for Interventional Trials-Artificial Intelligence
