# Supplementary material for: Investigating Data Diversity and Model Robustness of AI Applications in Palliative Care and Hospice: Protocol for Scoping Review
Source: JMIR Res Protoc. 2024 Oct 8;13:e56353. doi: 10.2196/56353 (PMC11496913; doi:10.2196/56353)
Supplement: Multimedia Appendix 1 [file resprot_v13i1e56353_app1.docx]

## Multimedia Appendix 1

1. Key Terms

| Palliative Care | Artificial Intelligence |
| --- | --- |
| 1. Palliative/Palliative Care/Palliation 2. Hospice* 3. Terminal Care/Terminally Ill* 4. Seriously Ill* 5. Terminal Phase/Terminal Stage 6. Inoperable/Incurable 7. End-of-Life/EOL 8. Life-Limiting 9. Actively Dying 10. Advance Care Planning | 1. Artificial Intelligence 2. Machine Learning 3. Deep Learning 4. Data Mining 5. Text Mining 6. Neural Network* 7. Natural Language Processing/NLP 8. Chatgpt 9. Generative AI 10. Generative Pre-trained Transformer 11. Reinforcement Learning 12. Learning Algorithm* 13. Integrated Learning 14. Sequence Learning 15. Network Analys* 16. Gradient Boost* 17. Boosting Algorithm* 18. Adaptive Boost*/Adaboost 19. Xgboost 20. Symbolic Regression 21. Supervised/ Unsupervised Learning 22. Random Forest 23. Decision Forest 24. Decision Tree 25. Support Vector Machine 26. Bayesian Network 27. Genetic Algorithm 28. Dimensionality Reduction 29. K-nearest Neighbour 30. Generative Adversarial Network* 31. GAN* 32. Generative Models 33. Transformers 34. Variational Autoencoders 35. VAES 36. Synthetic Data Generation 37. AI/ML Model |

Search strategies: Searched November 2, 2023, Firefox browser

PubMed 786

(“artificial intelligence”[tw] OR "Artificial Intelligence"[Mesh] OR “machine learning”[tw] OR machine-learning[tw] OR “deep learning”[tw] OR “deep neural learning”[tw] OR “data mining”[tw] OR datamining[tw] OR "Data Mining"[Mesh] OR “text mining”[tw] OR text-mining[tw] OR “neural network*”[tw] OR “natural language processing”[tw] OR “language process*”[tw] OR “large language model*”[tw] OR “NLP”[tw] OR “AI/ML”[tw] OR ChatGPT[tw] OR “Chat GPT”[tw] OR “generative AI”[tw] OR “generative artificial”[tw] OR “generative pre-trained”[tw] OR “pre-trained model”[tw] OR “conversational AI”[tw] OR “computational linguistic*”[tw] OR “computational intelligence*”[tw] OR “computer reasoning”[tw] OR “predictive analy*”[tw] OR “cognitive comput*”[tw] OR “text analy*”[tw] OR “iterative method*”[tw] OR “iterative reconstruction”[tw] OR “generative pre-trained transformer”[tw] OR “reinforcement learning”[tw] OR “learning algorithm*”[tw] OR "integrated learning"[tw] OR "sequence learning"[tw] OR "network analys*"[tw] OR "gradient boost*"[tw] OR “boosting algorithm*”[tw] OR “adaptive boost*”[tw] OR “AdaBoost"[tw] OR “XGBoost"[tw] OR "symbolic regression"[tw] OR "supervised learning"[tw] OR "unsupervised learning"[tw] OR “random forest"[tw] OR “decision forest"[tw] OR “decision tree"[tw] OR “support vector machine"[tw] OR “reinforcement learning"[tw] OR “Bayesian network"[tw] OR “Bayesian learning”[tw] OR “naïve bayes”[tw] OR “genetic algorithm"[tw] OR “dimensionality reduction"[tw] OR “K-nearest neighbour"[tw] OR “K-nearest neighbor"[tw] OR “adversarial network*”[tw] OR “GANs”[tw] OR “generative models”[tw] OR “transformers”[tw] OR “variational autoencoders”[tw] OR “VAEs”[tw] OR “synthetic data generation”[tw] OR “AI model”[tiab:~4] OR “AI models”[tiab:~4] OR “ML model”[tiab:~4] OR “ML models”[tiab:~4]) **AND** (palliative[tw] OR "Palliative Care"[Majr] OR palliation[tw] OR hospice*[tw] OR "Hospice Care"[Mesh] OR "Hospices"[Mesh] OR hospice*[tw] OR "Terminal Care"[Mesh] OR “terminal care”[tw] OR "Terminally Ill"[Mesh] OR “terminal illness*”[tw] OR “terminally ill*”[tw] OR “seriously ill*”[tw] OR “serious illness*”[tw] OR "terminal phase"[tw] OR "terminal stage"[tw] OR inoperable[tw] OR incurable[tw] OR unresectable[tw] OR “end of life”[tw] OR “end-of-life”[tw] OR “EOL”[tw] OR "last year of life"[tw] OR "actively dying"[tw] OR "Advance Care Planning"[Mesh] OR "advance care planning"[tw] OR “advance* care”[tw] OR “advance directive”[tw]) **AND** Eng[lang] **NOT** ("animals"[MeSH Terms] NOT "humans"[MeSH Terms]) **NOT** (preprint[ptyp] OR review[ptyp] OR “case reports”[ptyp] OR meta-analysis[ptyp] OR systematic[sb] OR "systematic review"[ptyp] OR editorial[ptyp] OR "pubmed books"[Filter] OR “letter”[ptyp] OR “comment”[ptyp] OR editorial[ti] OR letter[ti] OR comment*[ti] OR “systematic review*”[ti] OR “scoping review*”[ti] OR meta-analys*[ti] OR 'meta analys*'[ti])

Embase.com 1949

('artificial intelligence'/de OR 'machine learning'/de OR 'deep learning'/de OR 'deep neural learning' OR 'data mining'/de OR 'datamining'/de OR 'text mining'/de OR 'neural network*' OR 'natural language processing'/de OR 'language process*' OR 'large language model*' OR 'nlp' OR 'ai/ml' OR 'chatgpt'/de OR 'chat gpt'/de OR 'generative ai' OR 'generative artificial' OR 'generative pre-trained' OR 'pre-trained model' OR 'conversational ai' OR 'computational linguistic*' OR 'computational intelligence*' OR 'computer reasoning'/de OR 'predictive analy*' OR 'cognitive comput*' OR 'text analy*' OR 'iterative method*' OR 'iterative reconstruction'/de OR 'generative pre-trained transformer'/de OR 'learning algorithm*' OR 'integrated learning' OR 'sequence learning'/de OR 'network analys*' OR 'gradient boost*' OR 'boosting algorithm*' OR 'adaptive boost*' OR 'adaboost'/de OR 'xgboost'/de OR 'symbolic regression' OR 'supervised learning'/de OR 'unsupervised learning'/de OR 'random forest'/de OR 'decision forest' OR 'decision tree'/de OR 'support vector machine'/de OR 'reinforcement learning'/de OR 'bayesian network'/de OR 'bayesian learning'/de OR 'naïve bayes' OR 'genetic algorithm'/de OR 'dimensionality reduction'/de OR 'k-nearest neighbour'/de OR 'k-nearest neighbor'/de OR 'adversarial network*' OR 'gans' OR 'generative models' OR 'transformers' OR 'variational autoencoders' OR 'vaes' OR 'synthetic data generation' OR ai NEAR/4 model OR ai NEAR/4 models OR ml NEAR/4 model OR ml NEAR/4 models) **AND** ('palliative therapy'/exp OR palliative OR 'palliation'/de OR hospice* OR 'terminal care'/de OR 'terminal illness*' OR 'terminally ill*' OR 'seriously ill*' OR 'serious illness*' OR 'terminal phase' OR 'terminal stage' OR inoperable OR incurable OR unresectable OR 'end of life'/de OR 'end-of-life' OR 'eol' OR 'last year of life' OR 'actively dying' OR 'advance care planning'/de OR 'advance* care' OR 'advance directive'/de) **AND** [english]/lim AND [humans]/lim AND ([embase]/lim OR [medline]/lim OR [pubmed-not-medline]/lim) AND ('article'/it OR 'article in press'/it OR 'conference abstract'/it OR 'review'/it) **NOT** (preprint:ti OR review:ti OR “case report*”:ti OR meta-analys*:ti OR 'meta analys*':ti OR "systematic review":ti OR editorial:ti OR “letter”:ti OR “comment”:ti OR “scoping review*”:ti)

IEEE Xplore.com 455

(“artificial intelligence” OR “machine learning” OR machine-learning OR “deep learning” OR “deep neural learning” OR “data mining” OR datamining OR “text mining” OR text-mining OR “neural network*” OR “natural language processing” OR “language process*” OR “large language model*” OR “NLP” OR “AI/ML” OR ChatGPT OR “Chat GPT” OR “generative AI”) **AND** (palliative OR hospice OR “terminal care” OR “terminal illness*” OR “terminally ill*” OR “seriously ill*” OR “serious illness*” OR "terminal phase" OR "terminal stage" OR "actively dying" OR "advance care planning")

ProQuest Dissertations & Theses Global via Web of Science (Clarivate) 153

TS= (“artificial intelligence” OR “machine learning” OR machine-learning OR “deep learning” OR “deep neural learning” OR “data mining” OR datamining OR “text mining” OR text-mining OR “neural network*” OR “natural language processing” OR “language process*” OR “large language model*” OR “NLP” OR “AI/ML” OR ChatGPT OR “Chat GPT” OR “generative AI” OR “generative artificial” OR “generative pre-trained” OR “pre-trained model” OR “conversational AI” OR “computational linguistic*” OR “computational intelligence*” OR “computer reasoning” OR “predictive analy*” OR “cognitive comput*” OR “text analy*” OR “iterative method*” OR “iterative reconstruction” OR “generative pre-trained transformer” OR “reinforcement learning” OR “learning algorithm*” OR "integrated learning" OR "sequence learning" OR "network analys*" OR "gradient boost*" OR “boosting algorithm*” OR “adaptive boost*” OR “AdaBoost" OR “XGBoost" OR "symbolic regression" OR "supervised learning" OR "unsupervised learning" OR “random forest" OR “decision forest" OR “decision tree" OR “support vector machine" OR “reinforcement learning" OR “Bayesian network" OR “Bayesian learning” OR “naïve bayes” OR “genetic algorithm" OR “dimensionality reduction" OR “K-nearest neighbour" OR “K-nearest neighbor" OR “adversarial network*” OR “GANs” OR “generative models” OR “transformers” OR “variational autoencoders” OR “VAEs” OR “synthetic data generation” OR “AI model”NEXT/4 OR “AI models”NEXT/4 OR “ML model”NEXT/4 OR “ML models”NEXT/4) **AND** TS=(palliative OR palliation OR hospice* OR hospice* OR “terminal care” OR “terminal illness*” OR “terminally ill*” OR “seriously ill*” OR “serious illness*” OR "terminal phase" OR "terminal stage" OR inoperable OR incurable OR unresectable OR “end of life” OR “end-of-life” OR “EOL” OR "last year of life" OR "actively dying" OR "advance care planning" OR “advance* care” OR “advance directive”) **AND NOT** TI=(review OR “case report*” OR meta-analys* OR 'meta analys*' OR "systematic review" OR “scoping review*”)

Filters: **English** (Languages)

ClinicalTrials.gov 118

“artificial intelligence” **AND** (palliative OR hospice OR “terminal care” OR “terminal illness*” OR “terminally ill*” OR “seriously ill*” OR “serious illness*” OR "terminal phase" OR "terminal stage" OR "actively dying" OR "advance care planning")

Web of Science (Science Citation Index Expanded, Social Sciences Citation Index, Arts & Humanities Citation Index, Conference Proceedings Citation Index-Science, Conference Proceedings Citation Index-Social Sciences & Humanities, Book Citation Index– Science, Book Citation Index– Social Sciences & Humanities, Emerging Sources Citation Index, Current Chemical Reactions, Index Chemicus) 1151

AB=(“artificial intelligence” OR “machine learning” OR machine-learning OR “deep learning” OR “deep neural learning” OR “data mining” OR datamining OR “text mining” OR text-mining OR “neural network*” OR “natural language processing” OR “language process*” OR “large language model*” OR “NLP” OR “AI/ML” OR ChatGPT OR “Chat GPT” OR “generative AI” OR “generative artificial” OR “generative pre-trained” OR “pre-trained model” OR “conversational AI” OR “computational linguistic*” OR “computational intelligence*” OR “computer reasoning” OR “predictive analy*” OR “cognitive comput*” OR “text analy*” OR “iterative method*” OR “iterative reconstruction” OR “generative pre-trained transformer” OR “reinforcement learning” OR “learning algorithm*” OR "integrated learning" OR "sequence learning" OR "network analys*" OR "gradient boost*" OR “boosting algorithm*” OR “adaptive boost*” OR “AdaBoost" OR “XGBoost" OR "symbolic regression" OR "supervised learning" OR "unsupervised learning" OR “random forest" OR “decision forest" OR “decision tree" OR “support vector machine" OR “reinforcement learning" OR “Bayesian network" OR “Bayesian learning” OR “naïve bayes” OR “genetic algorithm" OR “dimensionality reduction" OR “K-nearest neighbour" OR “K-nearest neighbor" OR “adversarial network*” OR “GANs” OR “generative models” OR “transformers” OR “variational autoencoders” OR “VAEs” OR “synthetic data generation” OR “AI model”NEXT/4 OR “AI models”NEXT/4 OR “ML model”NEXT/4 OR “ML models”NEXT/4) **AND** AB=(palliative OR palliation OR hospice* OR hospice* OR “terminal care” OR “terminal illness*” OR “terminally ill*” OR “seriously ill*” OR “serious illness*” OR "terminal phase" OR "terminal stage" OR inoperable OR incurable OR unresectable OR “end of life” OR “end-of-life” OR “EOL” OR "last year of life" OR "actively dying" OR "advance care planning" OR “advance* care” OR “advance directive”) **AND NOT** TI=(preprint OR review OR “case report*” OR meta-analys* OR 'meta analys*' OR "systematic review" OR editorial OR “letter” OR comment* OR “scoping review*”)

Filters: **English** (Languages) and **Article** or **Proceeding Paper** or **Review Article** or **Early Access** (Document Types)
